# Depicting the anatomy of the gyral white matter: *ubi sumus? quo vadimus?*

**DOI:** 10.1093/braincomms/fcad265

**Published:** 2023-10-11

**Authors:** Guillaume Dannhoff, Phanindra P Poudel, Chacchu Bhattarai, Sneha Guruprasad Kalthur, Igor L Maldonado

**Affiliations:** CHU de Strasbourg, Strasbourg 67000, France; Inserm U1253, iBrain, Université de Tours, Tours 37032, France; Department of Anatomy, Manipal College of Medical Sciences, Pokhara 33700, Nepal; Department of Anatomy, Manipal College of Medical Sciences, Pokhara 33700, Nepal; Department of Anatomy, Kasturba Medical College, Manipal, Manipal Academy of Higher Education, Manipal 576104, India; Inserm U1253, iBrain, Université de Tours, Tours 37032, France; Department of Anatomy, Kasturba Medical College, Manipal, Manipal Academy of Higher Education, Manipal 576104, India

**Keywords:** cerebral cortex, white matter, autoradiography, neuroimaging, anatomy

## Abstract

A cerebral gyrus is made up of an external layer of folded cortex and an inner core of white matter. The architecture of the core has specific features that make it distinct from the white matter of the deep brain regions. Limited externally by the grey matter that covers the top of the gyrus and the neighbouring sulci, this gyral white matter is made up of a mix of fibre populations with multiple directions and destinations. The presence of densely packed fibres with multiple crossings, the proximity to the cortex and the existence of inter-regional and inter-individual variations make the task of depicting this microanatomy extremely challenging. The topic is, however, of paramount relevance for both fundamental and applied neurosciences. This fibre colocalization is crucial for the functional role of each cerebral region and is key to clinical manifestations in cases of parenchymal damage. As track tracing, imaging and dissection are based on different biological or physical principles, it is natural for their results to sometimes be different, but they are often complementary. As the amount of available information increases, it becomes fragmented due to the multiplicity of methods, target phenomena and studied species. In this scoping review, we present the key concepts and map the primary sources of evidence regarding identifying the fibre pathways that compose the gyral white matter, enabling the discussion of avenues for future research. The general pattern in which these pathways are distributed in the gyral white matter was detailed, and the main variations as a function of brain topography were explained and illustrated with typical examples.

## Teaser

Dannhoff *et al*. review the organization of gyral white matter, consisting of superimposed strata of association and striatal fibres surrounding a core of commissural and projection fibres. The proportion of each fibre type varies across brain gyri, relating to the functional role of each site.

## Graphical Abstract

**Graphical Abstract:**
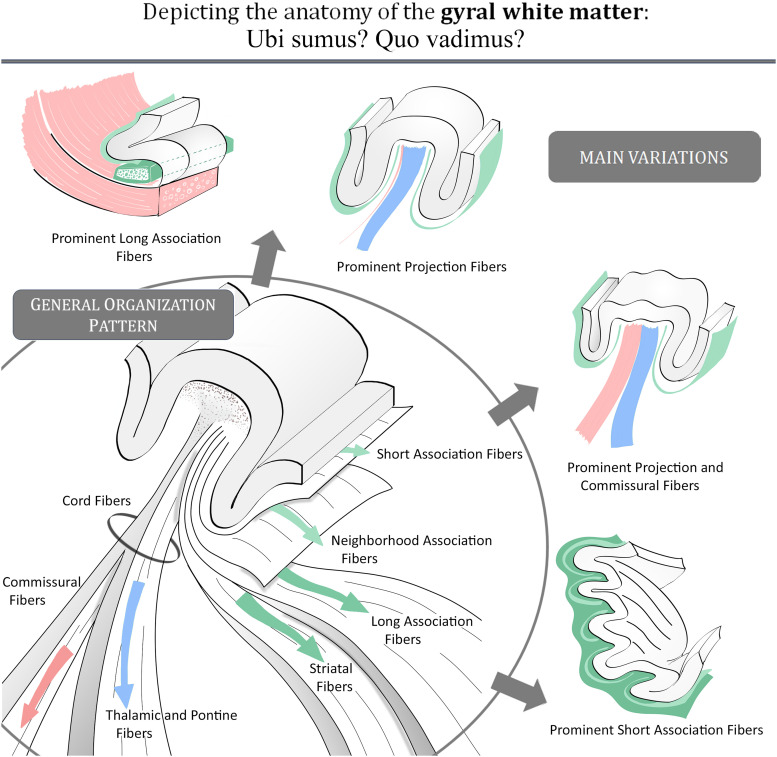

## Introduction

A cerebral gyrus combines an external layer of folded cortex and an inner white matter core. The architecture of the core has specific features that make it distinct from the white matter of the deep brain regions. Limited externally by the grey matter that covers the top of the gyrus and the neighbouring sulci, this gyral white matter (GWM) is made up of a mix of fibre populations with multiple directions and destinations. Short ‘U’ association and longer association, projection and commissural fibres are combined in variable proportions and reflect the specificities of each brain area.^1,2^

Significant methodological difficulties have hindered the in-depth study of this microanatomy. Among the main ones is the apparently homogeneous character of the white matter, which is usually depicted as amorphous by conventional anatomical or neuroimaging techniques. Moreover, the presence of densely packed fibres with multiple crossings, the proximity to the cortex and inter-regional and inter-individual variations make the task extremely challenging.^3-5^

The topic is, however, of paramount relevance for both fundamental and applied neurosciences. The aforementioned fibre colocalization is crucial for the functional role of each cerebral region in the context of the connectome and is key to the clinical manifestations in cases of parenchymal damage. For instance, the signs and symptoms of a stroke very frequently result from lesions that concern both the cortex and the underlying white matter. In the field of neuro-oncology, white matter architecture has been acknowledged as a significant influencer on the direction of tumour infiltration in cerebral gliomas.^6,7^

Untangling the anatomy of the GWM is, therefore, a research endeavour of great importance but difficult to access. Histology does not solve the problem since it shows the myriad of fibres but does not allow the operator to place them efficiently in the connectome. Among the recent efforts to decipher the subcortical white matter, the so-called mesoscopic techniques stand out, whether *in vivo* or *ex vivo*.^8^ In short, the main technical feature sought is the ability of the method to demonstrate tissue architecture on a scale that allows the acquisition of 3D data sets in large specimens (or the whole brain) but still depicts tiny white matter bundles. When this approach is insufficient, higher-resolution microscopic techniques may be applied to answer specific questions.

We have witnessed important technical advances in this line in the last three decades. Some may contribute to depicting the connective identity of brain areas in the midterm, but they are scattered across varied research fields. In non-human primates, radioisotope tracking and autoradiography have allowed important insights and have been in favour of a relatively consistent organization of the GWM for a given cerebral location.^2^ In human anatomy, fibre dissection gained much popularity and was combined with the surgical microscope.^9^ In *in vivo* imaging, both the acquisition and post-treatment of diffusion-weighted magnetic resonance images underwent significant advances, among which we can mention the increased magnetic field strength, diffusion gradient and angular resolution. In *ex vivo* imaging, the possibility of performing a prolonged acquisition opened a new avenue for future research, such as in the development of sequences, and significantly promoted the imaging of smaller structures.

The present paper is an effort to summarize this multi-source and sparse material on the GWM. Since track tracing, imaging and dissection are based on different biological or physical principles, it is natural for their results to differ in some aspects, but they are complementary. As the amount of information increases, it is fragmented due to the multiplicity of methods, target phenomena and studied species. Therefore, critical interpretation of new information is necessary and requires both neuroscientific and methodological knowledge.

In this scoping review, we present the key concepts and map the primary sources of evidence regarding identifying the fibre pathways that compose the GWM, enabling the discussion of avenues for future research.

## Materials and methods

A search was performed using MEDLINE/PubMed, Embase and Web of Science databases to identify articles in English on the structure of the white matter of the cerebral gyri in humans or non-human primates without restrictions related to publication date. A review protocol was drawn according to the Preferred reporting items for systematic reviews and meta-analyses statement for scoping reviews.^10^ The following descriptors were used: [architecture (title) OR anatomy (title) OR structure (title) OR morphology (title) AND (‘white matter’ OR fiber OR fibers) AND (gyrus OR gyri)]. Seminal publications on the topic were manually included regardless of whether they existed in the aforementioned databases. Publications that focused on the pathological aspects only, without any information on normal fibre anatomy, were excluded. For data synthesis, the authors used a shared computerized database with the publications retrieved and online documents, whose editions were discussed through regular (both online and presential) meetings.

As this review paper did not involve the collection or analysis of primary data from human subjects or animals, ethical assessment from an institutional review board was not applicable.

## Results

**Figure 1:**
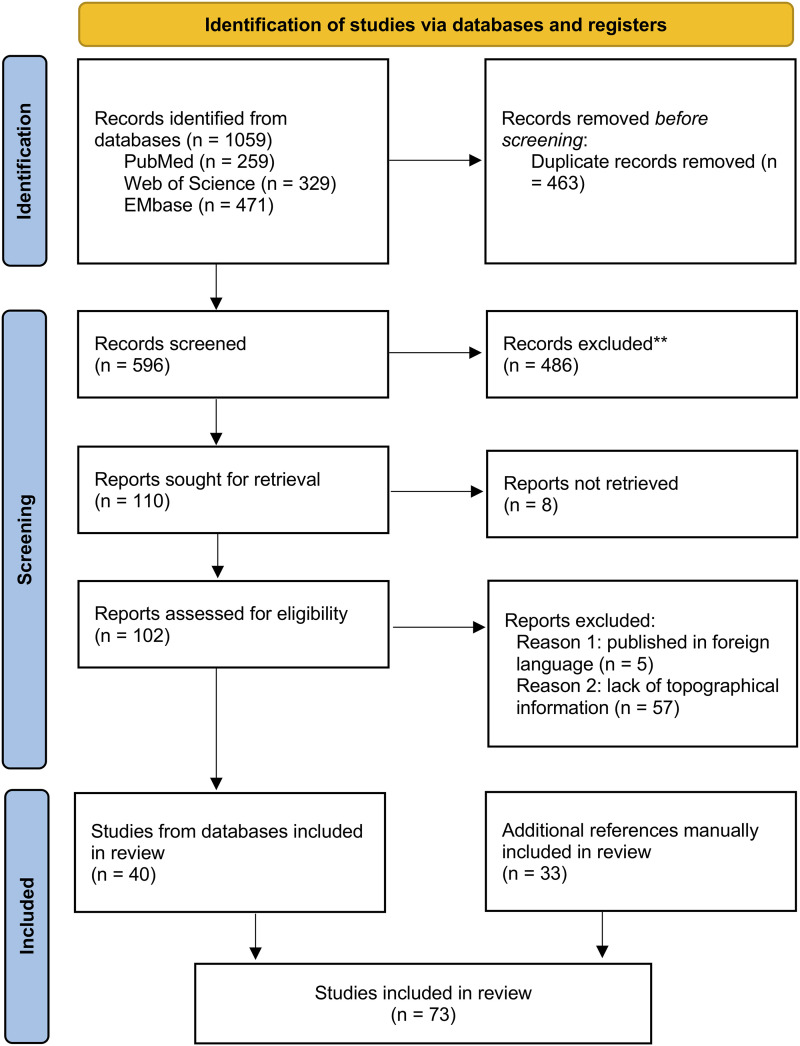
Preferred reporting items for systematic reviews and meta-analyses (PRISMA) flowchart, depicting the literature review process.

The initial search produced 596 different records after duplicates were removed. After analysing the titles and abstracts, we excluded 494 articles because they did not correspond to the study proposal, or they were not retrieved because they corresponded to referencing errors in the databases or were incompletely published. After examining the full texts of the 102 retrieved publications, we excluded 62 works that did not correspond to the study proposal. Another 33 documents were manually included after examination of reference lists and other sources, given their relevance to the topic. Finally, 73 publications were included in the review (Fig. 1).

The existing body of literature shows that the organization of the GWM is not homogeneous. Although a general pattern is acknowledged and applicable to most cerebral gyri, variations exist concerning the relative composition of terminations of projection, association or commissural fibres, even among regions of the same lobe. The following sections present these general aspects of the GWM organization, followed later by the particularities encountered in different brain regions.

### General aspects of the GWM architecture

Six layers and a fine myeloarchitectural network make up the grey matter of the neocortex. These layers establish simultaneous connections with a great variety of sites, including adjacent or distant gyri and even more distant centres in the neuraxis, such as the thalamus, basal ganglia, brainstem or spinal cord.^2^ A general arrangement is observed, with the relative position of fibres within the GWM being significantly associated with the group to which they belong.

**Figure 2:**
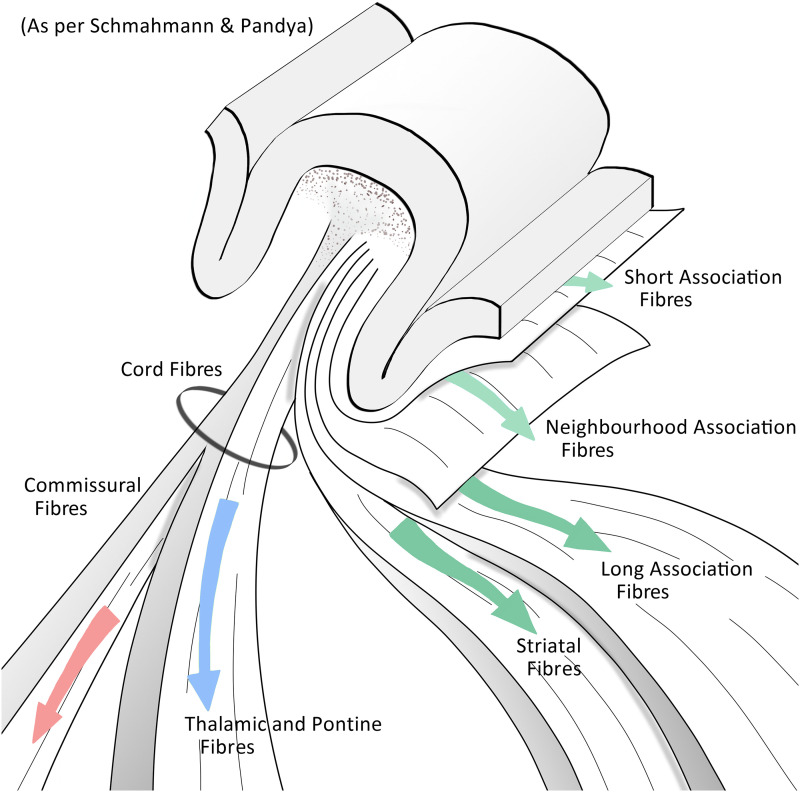
General pattern of organization of the gyral white matter according to autoradiography data (based on findings by Schmahmann and Pandya^2^).

Autoradiographic studies using radiolabeled tracers in non-human primates have revealed details of this relationship between anatomical position in the GWM and the nature of the connection with the other parts of the brain (Fig. 2). A first group is that of intra-hemispheric association fibres. Short ‘U’ association fibres linking adjacent gyri are present superficially under the cerebral cortex. Regional association fibres, destined for nearby regions within the same lobe, lie deeper than those shorter ones. Next, long association fibres interconnecting the gyrus to distant areas in the same hemisphere are still more deeply located. As a general rule, the more distant the cortical site interconnected by an association bundle, the more distant from the cortex the penetration of this bundle in the base of GWM will be.

According to the results of autoradiography, a second group comprises striatal fibres, which travel deeper than the association ones when they penetrate the base of the GWM. As they advance from the base to more superficial areas of the gyrus, significant intermingling (with the association fibres) is seen. These pathways connect the caudate nucleus, putamen and claustrum. A third group lies in the central axis of the GWM in the form of a well-defined core containing commissural and projection fibres that travel from and to deep regions of the cerebral hemisphere and beyond. In this subset, projections to the thalamus, brain stem and spinal cord are observed. Because of its central location, this group has been referred to in autoradiography studies as ‘the cord fibers’.^2^

Taking advantage of the effects of water crystallization through one or more freeze–thaw cycles, Klingler’s technique^11-13^ allows precise dissection of WM tracts rendered macroscopically discernable. In terms of the disposition of association fibres, the results of fibre dissection studies agree with those of autoradiography: short U fibres lie superficially to longer ones, and regional association fibres tend to get progressively longer as the operator advances to the depth.^14-19^ The short ‘U’ association fibre terminations seem more abundant at the top of the gyri than in their bases or the cortex of the adjacent sulci.^14,18^ This arrangement continues until a large fasciculus is reached (for instance, one of the longitudinal fasciculi).^11,16,20^

A few studies based on fibre dissection have taken an interest in depicting white matter connections converging in a specific gyrus in various regions of the human brain. These works brought valuable insights regarding fibres that are expected to be found within specific gyri within the frontal,^21-25^ temporal,^26,27^ parietal,^28-30^ and occipital lobe.^31-33^ Other works focused on topographical white matter aspects in defined regions of interest^19,34-38^ and bordering a surgical corridor^39,40^ or described the detailed anatomy of a specific tract with some topographic information.^41-47^ These studies give a global overview of the fasciculi converging to a given gyrus, but the intragyral organization of the elicited tracts is usually not depicted due to either methodological difficulties or the fact that the topic was not the subject of study.

**Figure 3:**
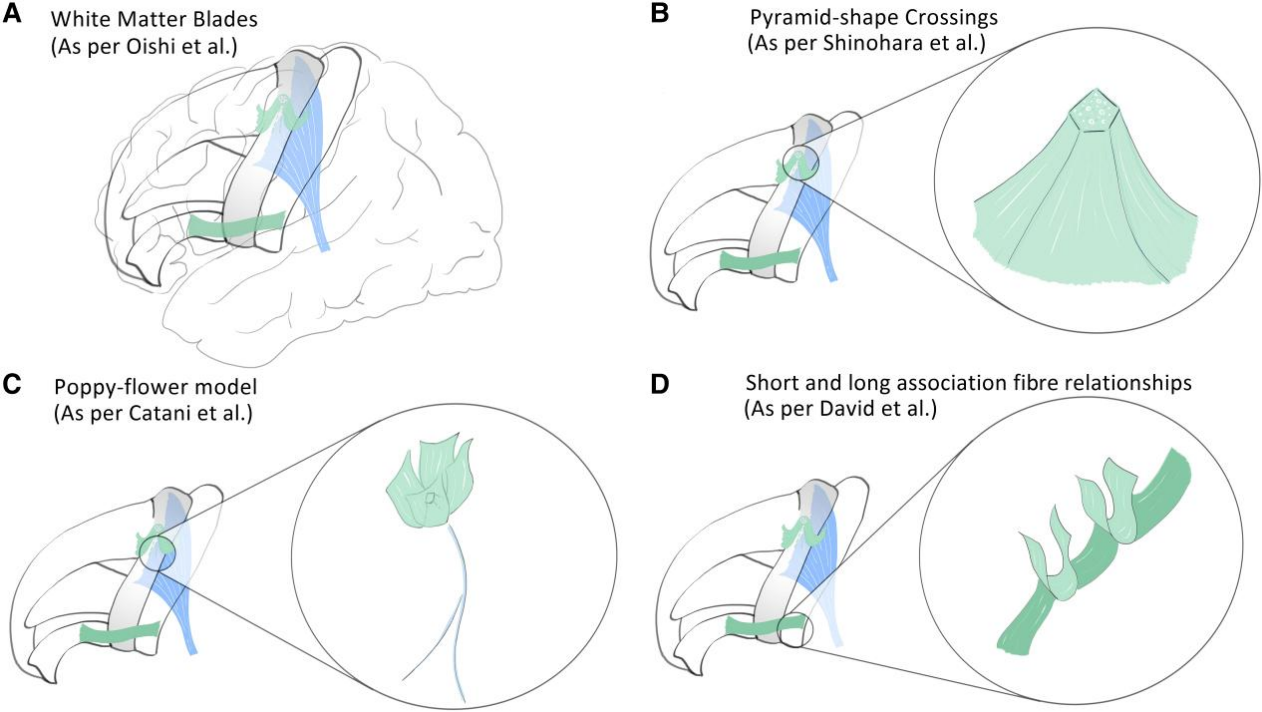
**Key concepts illustrating anatomical relationships within the gyral white matter, gathered from fibre dissection and tractography.** (**A**) White matter ‘blades’ described by Oishi *et al*.,^48^ representing consistent units of superficial white matter. Here are depicted the superior frontal, middle frontal, inferior frontal, pre-central and post-central blades. (**B**) Pyramid-shape crossings highlighted by Shinohara *et al*.,^18^ as converging points of U fibres in the superficial white matter. Here pyramid-shape crossings are depicted in the precentral-prefrontal region. (**C**) ‘Poppy flower’ model described by Catani *et al*.;^49^ illustrating a ‘flowering’ architecture of U fibre layers around a central core of projection fibres. (**D**) Intricate relations between short and longer association fibres, consisting in a potential confusion factor, as discussed by David *et al*.^41^

A significant contribution of fibre dissection is the capability to study the 3D shape of subcortical white matter after removing the cortical grey matter, which is rendered brittle by the preparation. The same applies to more complex structures that result from the convergence of fibre bundles. That is the case of the so-called ‘pyramid shape crossings’ (Fig. 3), resulting from the convergence of U fibres from various directions, which have been suggested as key structures for the intricate neural network and communication within and among the cerebral gyri.^18^

After removing the cortex, the exposed GWM takes the form of ridges that reproduce the gyral anatomy of the brain. Shinohara *et al*.^18^ detailed this arrangement using fibre dissection and highlighted the importance of exhuming the intragyral U fibres. The authors describe three types of junction areas according to their shape: three-way junctions, wide plate-like junctions and small ridge junctions. While intergyral U fibres pave the sulcal floors, intragyral U fibres are closer to the top of the gyrus and lie within the same white matter ridge. In the ridge junction areas, packages of intra- and intergyral U fibres converge at common sites, forming pyramidal shapes inside the GWM. Based on their trajectories and terminations, two general types of intergyral U fibres are possible, depending on whether they connect these convergence sites. This tridimensional organization of short association fibres combined with longer tracts colocated beneath one given gyrus gives rise to complex structures. These structures have been modelled by Catani *et al*.^49^ in the motor hand-knob region as a ‘poppy flower’ shape, with layers of U fibres diverging from the pyramidal core being analogous to the flower’s petals and the central cord fibres being analogous to its stem.

Regarding *in vivo* exploration of WM anatomy, diffusion-weighted imaging allows tractography to be performed via various algorithms. MRI tractography can confront anatomical data in humans with those of autoradiography in monkeys,^50^ and refine understanding of complex fibre tracts anatomy.^51^ To establish clear parcellation and systematic labelling, Oishi *et al*.^48^ used axonal fibre alignment information from diffusion tensor imaging to delineate the peripheral white matter, considering its relationships with the cortex and the deep white matter. The authors used the population-averaged tensor map to identify common features across subjects. The concept of a blade-like morphological unit (or simply ‘blade’) was proposed.^48^ Nine such large units were identified, which were further parcellated into 21 subregions based on the cortical anatomy. The subparcellated regions in each blade have been referred to as ‘gyrus name WM’. Four large groups of intergyral U fibres were described as central for interblade connections and identified as frontal (between the superior and inferior frontal blades), frontocentral (between the middle frontal and the precentral blades), central (between the pre- and post-central blades) and parietal (between the superior parietal and the parietal–temporal blades). Another study on comparative anatomy from the same group suggested that the U fibres interconnecting the superior parietal to the occipital blade are present in humans but not in the macaque brain.^52^

### GWM architecture in brain areas

The general pattern described above varies with the topography of the studied gyrus. The main situations and those for which evidence from the literature is available are described below. The study of the short association bundles formed by clusters of U-shaped fibres with diffusion imaging is a field that has notably developed in recent years and provides a clear illustration of how the organization of superficial white matter is prone to intra- and inter-individual variations. Numerous surface bundles, ranging from 29 to 50 per hemisphere, have recently been described in an ongoing effort to systematize.^53-55^

Among the current hypotheses regarding cerebral gyrification are the differential growth of cortical regions (driving cortical folding under cranial constraint)^56^ and axonal tension-based theories either pushing out or pulling in.^5,57^ Besides the logical role of cortical expansion leading to folding, the role of axonal wiring in determining cortical folding is likely. It has been demonstrated that the maximum density of axonal fibres in humans and non-human primates is found in the gyral region compared to the sulci,^5^ which is concordant with most observations in fibre dissection and diffusion imaging. Moreover, the complexity of the gyri’s connections has been significantly linked with fibre densities, as three-hinge gyri show much higher fibre densities than two-hinge gyri.^58^ These findings suggest a potential role for fibre connections as an inducing factor in gyrification schemes, underscoring the close relationship between surface anatomy variations and underlying white matter structure.

Short-range connectivity is particularly relevant when taking an interest in the morphology of GWM, as it represents around 60% of total white matter volume,^59,60^ a major factor for local variations of GWM. Examining data sets of 90 subjects from the Human Connectome Project, Bodin *et al*.^63^ identified *plis de passage* linking adjacent gyri, formerly described by Gratiolet and Broca, as cortical landmarks^61,62^ associated with regions of accrued short-range connectivity. Bajada *et al*.,^64^ studying fibre length profiles, demonstrated a concentration of shorter fibres (<40 mm) in primary cortices, notably the somatomotor cortex.

As a factor that may impact the understanding of short-range connections, variation in the brain’s gyrification pattern must be taken into account, which prevents conclusions from being drawn from isolated cases and imposes the study of many subjects. For this reason, some cerebral regions are more susceptible to this type of assessment than others. The central sulcus is almost always continuous and relatively deep, leading to lower structural inter-subject variability in motor areas.^65^ In the central region, pre-central and post-central GWM blades are linked by U fibres for their entire length.^52^ This consistent line of U-shaped fibres is situated between two large contingents of projection fibres that belong to the corticospinal tract anteriorly and to the thalamic radiations of the ventral posterolateral nucleus of the thalamus posteriorly.

Unraveling local variations of the GWM organization also brings valuable insights into the functional specificities of a given region. Such inferences are greatly illustrated by pre- and post-central gyri—the terminations of prominent projection fasciculi—where U-shaped fibres adopt the functional arrangement of Penfield’s homunculus.^49^ This short fibre connective system linking pre-central and post-central gyri might modulate the clinical outcome following subcortical stroke, a concept associated with variations in the shape of the central sulcus,^66,67^ which is itself directly correlated with U-shaped fibre anatomy. Some correlations between morphological landmarks and function localization were proposed in line with these theories. That is the case of the *pli de passage moyen* described by Broca, which has been associated with accrued short-range connectivity between the hand motor activation and the hand sensory regions.^63,68,69^ The association between manual dexterity and the structural features of these bundles in the hand area indicates their potential role in fine motor control.^70^ In pathological conditions, one could finally easily infer, in the light of these descriptions, how the clinical signs of a Jacksonian seizure would progress along this superficial network of the somatomotor region, as already suggested in early anatomical works.^71^

Using diffusion imaging and a technique called unsupervised clustering, Pron *et al*.^72^ identified five major bundles crossing the central sulcus in both the left and right hemispheres. In accordance with previous descriptions, the authors described a consistent organization of these bundles according to the somatotopy of the central region: one bundle for the tongue (ventral end), three for the hand and one for the foot region (dorsal end).^54,55,72,73^ Precise systematization of this anatomy, however, has been prone to discussion, with a possible subdivision of the ventral interconnections in two subbundles,^49^ as a possible interindividual variation.^55,72^ Furthermore, variations in the position of each bundle may be related to functional aspects. In this line, a dorsal translation of the hand bundle has been suggested to be associated with handedness.^55,72,74^

Another example of a consistent anatomical relationship between adjacent projection and short association fibres with functional implications is that observed in the primary visual cortex. The medial occipital lobe, made of the cuneus and lingual gyrus, is implicated in visual processing. These gyri show a large contingent of short association fibres around the calcarine fissure, namely, the stratum calcarinum of Sachs, which is likely to be a key structure in transmission from primary visual areas to associative areas.^15,75^ Moreover, these fibres support communication between streams within the visual system, namely, the dorsal ‘where’ recently renamed ‘how’ pathway^76-78^ and the ventral ‘what’ pathway. Focal damage to these white matter pathways may lead to selective impairments of the complex visual processing such as the associative visual agnosia defined by Lissauer.^75,79^

A particular case is that of the superficial white matter around the lateral (Sylvian) fissure. The extreme capsule ensures continuity between the subcortical opercular white matter and that of the insula, an arrangement similar to that of the subcortical white matter around the shallower sulci on the brain surface. Operculo-opercular connections through the extreme capsule have been characterized in the Rhesus monkey thanks to autoradiography. A clearly continuous layer of white matter extending from the extreme capsule to both opercula and vice versa is observed in human dissection and neuroimaging studies, even if the actual content of opercula–opercular pathways is not detailed.^80^ More superficially, short association fibres are observed and follow the sulcogyral anatomy of the insula and the inner opercular surface (e.g. sub-opercular, sub-triangular and transverse gyri).^49,73^

The GWM arrangement in the insula is marked by accrued short-range connectivity. Being the insula situated at the depth of the Sylvian fissure, this organization rich in short insulo-insular tracts is completed by insulo-opercular connections. The frontoparietal and temporal opercula are regions of prominent association fibre, bearing the colocalization of U-fibres and converging and passing long fibres, primarily concentrated in the language network. As a result, the frontal operculum concentrates short association fibres originating in the pre-central gyrus, middle frontal gyrus and upper anterior insula. This short-range connectivity is reinforced by the intralobar frontal aslant tract and by the anterior terminations of the SLF-III, itself a part of the SLF/AF complex. The role of the insula in language production is still a matter of debate but may result more from this particular connectional position than from a dedicated cortical role.

A particular variant of the GWM arrangement is the prominence of longitudinal long-association fibres inside the GWM. The component of projection and commissural fibres is consequently smaller. The cingulate gyrus, part of the limbic lobe, is the most typical example. It is implicated in a wide range of functions, such as memory, emotion, motivation, higher cognition and motor control. The cingulum bundle runs longitudinally within this gyrus, providing multiple interconnections to areas located inside or outside the limbic lobe.^81,82^ The general aspect of the core white matter is then that of a compact longitudinal fasciculus, although it actually comprises several subcomponents with different terminations and functions and an important contingent of diencephalic projections to the limbic lobe. U fibres surround this core, connecting the cingulate cortex with the orbitofrontal, prefrontal, parietal and temporal cortices, but are absent in the portions where the cingulum makes direct contact with the corpus callosum without the interposition of the cerebral cortex. Abnormality in the morphology of these U fibres has been implicated in neuropsychiatric disorders such as autism spectrum disorders.^83^ Such prominence of longitudinal fibres in the GWM is observed to varying extents in other locations, such as the anterior portion of the parahippocampal gyrus (also penetrated by the cingulum),^33^ and the *limen insulae* (due to the presence of uncinate and inferior fronto-occipital fasciculi).

An almost opposite arrangement is observed along the upper border of the cerebral hemisphere. That is the case for the superior frontal, pre-central and post-central gyri (superior extremity), paracentral lobule and superior portion of the precuneus. The robustness of the contingent of projection and commissural fibres originating from the *corona radiata* and dorsal callosal radiations in those regions forms a compact central scaffolding with a vertical orientation. Although these two components are intermingled, both diffusion imaging and fibre dissection agree that projection fibres predominate laterally and commissural callosal fibres medially. Short and regional association fibres are abundant on either side of this barrier. Some diffusion imaging and autoradiography studies favoured a robust long longitudinal fasciculus crossing all of these gyri. This arrangement has been the subject of debate in fibre dissection and other diffusion imaging studies in humans regarding both its appearance and exact path.^84,85^ Long longitudinal pathways have been, however, demonstrated in a more ventral (cingular or paracingular) location.^81,86,87^

## Discussion

In the present study, we performed a scoping review to identify key concepts and current sources of evidence regarding the architecture of GWM in humans and non-human primates so that avenues for future research can be discussed. Historically, two fundamental techniques have allowed studies of the composition of GWM: autoradiography in non-human primates and fibre dissection in humans. Emerging mesoscopic techniques, such as *ex vivo* high-field magnetic resonance imaging, are promising, but studies employing these techniques in the superficial white matter are still scarce. The lack of precise anatomical descriptions and the lack of systematization of GWM in humans contrast sharply with the topic’s functional relevance and potential clinical applications.

**Figure 4:**
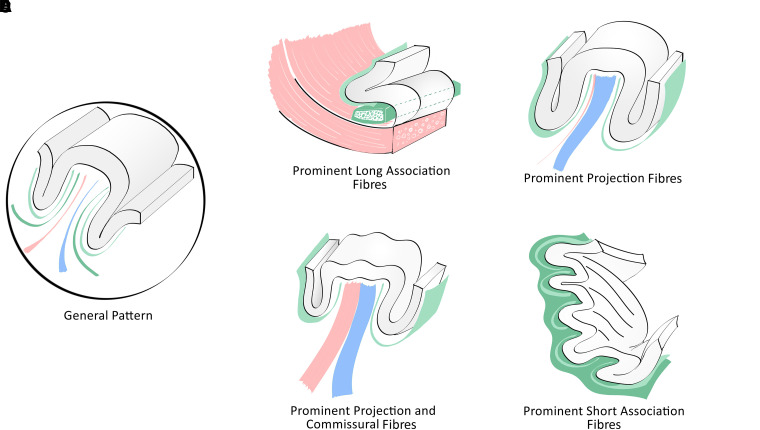
**General pattern of the organization of the gyral white matter and main variants according to specific topographies and differences in the proportions of its components.** Association fibres are depicted in blue, projection fibres in green and commissural fibres in pale red. (**A**) General pattern. (**B**) Prominent long association fibres. In this situation, a robust association fasciculus occupies most of the gyral white matter. Typical examples are the cingulate gyrus, the limen insulae and the parahippocampal gyrus. (**C**) Prominent projection fibres. In this situation, core projection fibres are particularly prominent. Typical examples are the pre-central and post-central gyri. (**D**) Prominent projection and commissural fibres. This organization is mostly observed in gyri located along the superior border of the cerebral hemisphere, which are rich in terminations of projection fibres from the corona radiata and callosal radiations. Typical examples are the superior frontal gyrus and the paracentral lobule. (**E**­) Prominent short U association fibres. In this situation, intergyral fibres predominate. Typical examples are insular gyri and most secondary gyri.

**Figure 5:**
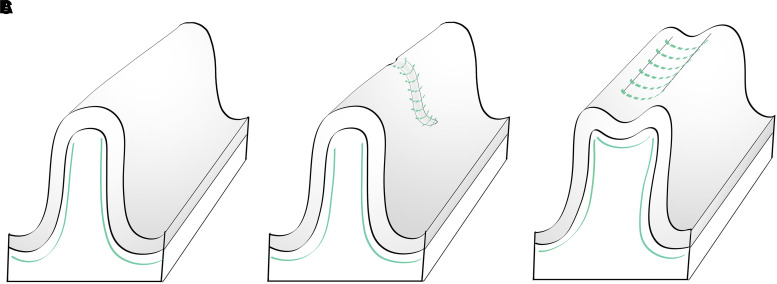
**Impact of secondary sulci and gyri on the disposition of short ‘U’ association fibers and the richness of intragyral bundles.** (**A**) Smooth gyral surface. (**B**) Presence of shallow secondary sulci. A typical example is the medial aspect of the superior frontal gyrus. (**C**) Presence of shallow longitudinal sulci. This arrangement is frequently observed in the medial aspect of the cingulate gyrus and the lateral aspect of the middle frontal gyrus.

A general pattern for the organization of the GWM has been proposed based mainly in autoradiography data from the Rhesus monkey brain. This pattern is, however, subject to variations in the proportion of the different GWM subcomponents (projection, commissural, long and short association fibres). In the human telencephalon, more drastic changes may be observed related to one or more of the three following phenomena: (i) prominence of one or two subcomponents, (ii) convergence of short association bundles (often associated with confluent gyri or *plis de passage*); (iii) presence of anatomically evident intragyral short association bundles. In these situations, the general pattern can be absent on one or both banks of the gyrus. In an effort to synthesize this information, we propose a classification of the human GWM structure based on these patterns, which is detailed in Fig. 4. In the specific case of the presence of secondary sulcus and gyri—minor anatomical accidents that do not alter the overall appearance of the gyrus—short association bundles occupy subcortical white matter, as they do for the major gyri. This situation is schematized in Fig. 5.

The great reliance, simplicity and applicability of fibre dissection in the study of the 3D anatomy of the brain led it to be designated by some authors as the gold standard of white matter study from an anatomical perspective. Though of great morphological interest, this technique has the disadvantage of being destructive. Hence, each dissection step deprives the operator of a quantity of information from the previous step. Moreover, aggressive dissection—especially when metallic microinstruments are used—can generate spurious tracts delivering artifactual anatomical data. These limitations are particularly strong regarding the GWM, as it may have been injured during the first dissection steps. Indeed, superficial fibres are often sacrificed hastily in order to quickly shift the focus to more obvious deep tracts in most anatomical papers. Some authors have proposed digitization techniques enabling the storage of 3D data from dissection, allowing the possibility to easily ‘look behind’ and contextualize dissection in light of the superficial layers. In 2014, Zemmoura *et al*.^88^ reported a method (FibraScan) in which *in vivo* or *ex vivo* MRI data sets could be compared to the results of fibre dissection. With the help of a surface laser scanner, which is applied iteratively to the cerebral specimen after each dissection step, the operator can follow, manually segment and reconstruct the dissected white matter tracts in the reference space of the MRI. This method allows the comparison of dissection to *in vivo* and *ex vivo* tractography. Since it requires optimal exposition of the target area to the surface scanner, the depth of the sulci and the secondary gyri are less accessible to this technology.

Diffusion-weighted imaging provides non-invasive access to structural connectivity *in vivo*.^89,90^ However, since the method is indirect, it has some limitations, especially near the crossings and the cerebral cortex.^91^ Performing accurate tractography can be challenging in both situations, as the anisotropy fraction is typically reduced. Moreover, classical deterministic tractography based on diffusion tensor imaging can be considered reductionist since it identifies one preferential direction of diffusion inside a relatively large portion of space (the voxel), which may contain different populations of fibres.

Several mathematical models have been proposed to overcome this limitation in the last two decades. Some model different fibre populations within the same voxel, estimating orientation distribution functions and enabling better mapping of crossing fibers.^92^ Q-ball imaging^93,94^ and generalized q-sampling imaging^95^ are among the main approaches in this group, which are also particularly relevant in pathological conditions exhibiting complex diffusion patterns.^91^ Spherical deconvolution methods^96,97^ benefit from deconvolution kernels to resolve fibre orientations, bearing a high potential for addressing crossing fibers.^98^ In all cases, however, fibre anatomy is deduced as a function of diffusion directions and indirect character that, added to the complexity of the tissue weave, claims for validation studies against ground truth anatomical data.

In the detailed study of brain anatomy, *ex vivo* MRI has some advantages over *in vivo* MRI. *Ex vivo* ultra-high-field MRI combines ultra-powerful gradients (11.7 T) with virtually unlimited acquisition time and the absence of physiological, motion or thermal noise. This technique can produce T_2_-weighted and diffusion-weighted images in mesoscopic resolution, as high as 100 µm of voxel size or smaller. This increase in resolution makes it possible to discern less visible structures in clinical imaging, but they do not present with the same signal as *in vivo* MRI. There are several reasons for this change, the main one being that the anatomical specimen is generally fixed in formalin, which modifies (reduces) relaxation times. To overcome these concerns, the interest in stronger magnetic fields in *ex vivo* imaging is undeniable.

Recent microscopic methods that are not (or less) applicable to the study of the whole brain are promising for investigating specific questions, especially in complex regions or those for which the techniques mentioned above were deemed insufficient. That is the case with optical coherence tomography, a method developed by Fujimoto *et al*. in 1991 to study the eye. In recent years, it has been applied to rodent and human brains. This imaging technique relies on the optical properties of the tissues—mainly fibres and neurons—and has a resolution of a few micrometers. The images exhibit excellent contrast, reliably differentiating cortical layers, nuclei and tracts. Since optical coherence tomography can only probe a few hundred microns in depth, Magnain *et al*.^99^ suggested coupling the system to a vibratome to allow for iterative acquisition.

Light-sheet microscopy is another promising tool for reconstructing the 3D architecture with micron-scale resolution. This approach allows for the 3D mapping of the neuronal organization of a slab of 10 × 10 × 0.05 cm^3^ in a few hours. The sample is illuminated with a thin light sheet, and the fluorescence emission is collected along a perpendicular axis. It confines the excitation light to the focal plane, allowing optical sectioning and imaging with cellular resolution. To obtain a good reconstruction, light-sheet microscopy requires the sample to be transparent or coupled with clearing procedures. The SWITCH immunostaining technique with the refractive index matching medium TDE (2,2′-thiodiethanol) was recently combined with light-sheet microscopy, obtaining an efficient method for larger volumes of human brain parenchyma, an advance that creates the possibility of studying the 3D architecture of a tissue fragment, including fibre bundles within their natural environment.^100,101^

3D-polarized light imaging (3D-PLI) is another neuroimaging technique that has opened up new avenues to study fibres in post-mortem brains.^102,103^ 3D-PLI does not require staining or labelling because it uses an intrinsic tissue property, birefringence, to modify the polarization state of the light. 3D-PLI capitalizes on the birefringence of myelinated and unmyelinated fibres, determining their spatial orientation at microscopic resolution. Based on the non-linear registration of serial fibre orientation images, virtual 3D models of fibre structures are created. As a microscopic (and not mesoscopic) technique, 3D-PLI resolution is exceptionally high, which generates large data sets. It is currently appropriate for the study of tissue samples but less suitable for the whole length of a long fasciculus or an entire human brain. It comes up against the availability of these methods, such as brain optical coherence tomography and light sheet microscopy, which are limited to a small number of expert centres.

As the organization of the GWM is a reflection of a gyrus role in the brain circuitry, it has immediate clinical applications, ranging from understanding mechanisms at stake in normal aging to identifying abnormal connectivity or even clarifying plasticity mechanisms. Pathways for future research in these domains are numerous. Some psychiatric syndromes have been correlated with alterations of the superficial white matter network, particularly those linked to emotion regulation in bipolar disorder.^104-106^ Abnormalities in superficial connectivity have been described in schizophrenic patients, ranging from functional alterations^105,107^ to focal structural differences, such as an increased density in specific brain regions.^108,109^ Short-range connectivity abnormalities have been identified in patients affected by autism spectrum disorder as well, notably as a potential correlate of social deficits.^110^

Several clinical and radiological observations suggest a role for superficial white matter in cognitive processes. Alterations of superficial white matter have been associated with cognitive decline in aging patients^111^ and as a marker of neurodegenerative disorders such as Huntington’s disease and in the asymptomatic phase.^112^ Extensive damage to the superficial white matter has been identified in patients suffering from auto-immune encephalitis with incomplete cognitive recovery.^113^ The characterization of the GWM could thus lead to a better understanding of the functions at stake and the identification of preclinical markers.

Thanks to recent advances in unraveling brain connectomics, understanding white matter functional anatomy has become mandatory for the modern brain surgeon in order to optimize surgical approaches, bearing in mind a ‘zero-footprint’ ideal.^114-116^ Historically, the scarcity of anatomical data has regrettably led most neurosurgeons to plan cortical incisions without considering superficial fibres. A better understanding of the complex GWM anatomy could lead to a rethinking of *trans*-parenchymal approaches and tailor cortical incisions to maintain the integrity of both superficial and deep white matter pathways^19,117^ and favour neuroplasticity mechanisms.

The advent of increasingly complex structural connectivity models—though scientifically appealing—may hinder accessibility and applicability in everyday practice. Since it is paramount for clinicians and researchers to remain able to maintain a mental picture of the white matter anatomy, our graphical classification of GWM is a convenient way to approach structural connectivity from a topographic perspective. As clinicians commonly deal with superficial brain landmarks in day-to-day practice, this classification is intended to provide a simple representation of the general macroscopic organization of the underlying structural connectivity, which can be used in many contexts. Among the main examples is the planning of surgical approaches to the cerebral parenchyma and the understanding of main pathways at play in a pathological process affecting a gyrus.

Although the present work broadly addresses the under-evaluated subject of GWM, it has some limitations. It suffered from the paucity of robust data currently available in the literature. Many papers included in the present study were not aimed directly at describing GWM but did contain important information. This status as an indirect subject can lead to more difficulty in detecting hidden information. For this reason, the research equation included a wide range of terms to screen as many articles as possible. The abstracts and contents of the selected articles were carefully screened, and references were checked to target any significant contribution. Being the first review on this topic, one of its strengths is its interdisciplinary character that favours cross-fertilization of the results obtained with different research techniques.

## Conclusion

According to the results of the present review, the gyri of the human brain are key topographical arrangements of the convergence of white matter tracts, with a common overall organization that appears similar to that of non-human primates. However, current data already point out the multitude of regional adaptations in specified regions of the cerebrum, bearing enlightening morphofunctional correlations. The most drastic changes in the general pattern are fundamentally due to differences in the proportion of fibres that populate this gyral white matter. Variants are numerous but most often fall into one of the following categories: prominent association fibres, prominent projection fibres, prominent projection and commissural fibres and prominent short association fibres. In order to better understand this complex hodotopical organization, a thorough understanding of this tridimensional colocalization of white matter fibres is paramount.

Although several techniques are currently available for studying white matter anatomy, they suffer from methodological limitations, making the detailed study of superficial white matter difficult, especially *in vivo*. Recent technological advances offer scarce data but raise hopes for thorough studies in the years to come. The clinical importance of the GWM thenceforth hints at great interest and will undoubtedly benefit from such detailed anatomical studies, with the promise of unraveling a whole new aspect of neuroanatomy and brain connectomics.

## Data Availability

Data sharing is not applicable to this article as no new data were created or analysed.
